# Undetected Weight Loss Associates With Upstaging in Cancer Patients

**DOI:** 10.1002/jcsm.70266

**Published:** 2026-04-08

**Authors:** L. Anne Gilmore, Evie Y. Dunsky, A. Jacob Miller, Christian M. Alvarez, Santiago Olaechea, Brian W. Gilmore, Chul Ahn, Puneeth Iyengar, Rodney E. Infante

**Affiliations:** ^1^ Center for Human Nutrition University of Texas (UT) Southwestern Medical Center Dallas Texas USA; ^2^ Department of Clinical Nutrition University of Texas (UT) Southwestern School of Health Professions Dallas Texas USA; ^3^ Department of Computer Science and Engineering University of North Texas Denton Texas USA; ^4^ School of Public Health University of Texas Southwestern Medical Center Dallas Texas USA; ^5^ Department of Radiation Oncology Memorial Sloan Kettering New York New York New York USA; ^6^ Harold C. Simmons Comprehensive Cancer Center University of Texas (UT) Southwestern Medical Center Dallas Texas USA; ^7^ Department of Internal Medicine University of Texas (UT) Southwestern Medical Center Dallas Texas USA

**Keywords:** cancer cachexia, diagnosis, gastrointestinal cancer, non‐small cell lung cancer, unintentional weight loss

## Abstract

**Background:**

Unintentional weight loss (UWL) is the primary diagnostic parameter for cancer cachexia in the clinic. Prompt identification of UWL can lead to earlier diagnoses and interventions for cancer. This study investigates the frequency and timing of UWL documentation and diagnoses in a cohort of cancer patients with measured UWL and sought to understand how recognition of UWL is related to stage of disease at cancer diagnosis.

**Methods:**

A retrospective cohort of adult gastrointestinal and non‐small cell lung cancer patients with measured UWL was evaluated. Body weight histories were manually reviewed for UWL. Patients with intentional weight loss, weight loss related to fluid status or weight loss with unclear intention were excluded. Records were assessed for the use of UWL‐related International Classification of Diseases codes and documentation of UWL. ULW was considered missed if it was not documented or diagnosed by a healthcare provider. Associations between weight loss over time, documentation and/or diagnosis of UWL and stage of disease at cancer diagnosis were investigated through repeated measures mixed models and univariate and multivariate tests.

**Results:**

In total, 374 patients (24%) met the definition of UWL with an average weight loss of 8.6% ± 0.3%. UWL was missed in 161 (43%) patients with cancer cachexia. The odds of undetected UWL were greater in patients < 65 years old, with NSCLC, with obesity and who were White. In the 213 patients with recognized UWL, the documentation of UWL occurred approximately 7 months after their initial weight loss. The magnitude of weight loss was associated with cancer stage at cancer diagnosis (*p* < 0.0001). Patients who experienced ≥ 7.5% UWL pre‐cancer diagnosis had a median cancer stage of 3 whereas patients who experienced < 7.5% UWL had a median cancer stage of 2 at cancer diagnosis (*p* < 0.0001). Relative to patients with ≥ 7.5% weight loss, the < 7.5% weight loss group had 73.7% more patients with Stage 1 disease (*p* < 0.0001) and 32.5% fewer patients with Stage 4 disease (*p* = 0.0006) resulting in median cancer stage increasing from Stages 2 to 3.

**Conclusions:**

UWL was missed in 43% of cancer cachexia patients despite 8.6% ± 0.3% body weight loss prior to cancer diagnosis. When recognized, documentation of UWL did not occur until 7 months after weight loss initiation. A greater magnitude of UWL was associated with more advanced disease at cancer diagnosis. Recognition and timely diagnosis of UWL will increase the percentage of patients with curable, early‐stage disease.

## 1 Introduction

Cancer cachexia is a progressive, multifactorial syndrome associated with metabolic alterations and systemic inflammation leading to anorexia and loss of fat and lean mass [1, 2]. The prevalence of cachexia at cancer diagnosis, defined by unintentional weight loss (UWL) of ≥ 5% over a 6‐month time frame as per the international consensus definition [3], ranges from 30% to 80% in patients with solid malignancies, particularly gastrointestinal cancers including pancreatic, gastro‐oesophageal and luminal cancers and lung cancers [4, 5]. Why some cancers cause cachexia and others do not is unknown. Recent findings suggest genetic mutations within the malignant cells could drive this process [6]. As cachexia progresses, it becomes less responsive to pharmacologic and nutritional interventions and has a significant impact on patient morbidity and mortality, making it critical to identify early [7, 8, 9]. Independent of other clinical factors, cancer‐associated weight loss is thought to be responsible for 30% of all cancer‐related deaths and is associated with reduced survival [9, 10, 11].

Although UWL is the primary diagnostic parameter for cancer cachexia in the clinic, there has been limited granularity regarding weight loss trajectories prior to a cancer diagnosis. To quantify the timing and extent of cancer‐associated weight loss in patients with gastrointestinal or non‐small cell lung cancer (NSCLC), we previously evaluated the kinetics of weight change over the 12‐month periods preceding and after cancer diagnosis. Our data revealed significant weight loss initiated as far back as 12 months prior to a cancer diagnosis [12, 13]. Initiation of weight loss almost a year prior to cancer diagnosis led us to investigate if clinicians recognized the UWL prior to the patient's cancer diagnosis. Here we present a retrospective cohort of 376 cancer patients with measured UWL in the year prior to a GI or NSCLC cancer diagnosis.

## 2 Methods

### 2.1 Population Cohort and Data Acquisition

This study was approved by the UT Southwestern Institutional Review Board prior to data acquisition (protocol STU 092013‐028). This cohort includes patients diagnosed with gastrointestinal (GI; colorectal, gastroesophageal, hepatic, biliary or pancreatic) or NSCLC at UT Southwestern Medical Center between May 2005 and December 2019. Patients were required to have at least one body weight in the 12 months prior to cancer diagnosis and one body weight measure at diagnosis to assess pre‐cancer diagnosis weight loss. Body weight data were assessed for weight loss and manual chart reviews were completed to determine weight loss intention. The final analysable cohort included GI cancer and NSCLC patients with UWL in the 12 months prior to cancer diagnosis. Clinical data were automatically and manually extracted from the electronic health record (EHR) and institutional tumour registry.

### 2.2 Organization of Body Weight Data

All body weights recorded in the patient's EHR pre‐cancer diagnosis were extracted and longitudinally organized in 3‐month intervals 1‐year pre‐cancer diagnosis. The body weight assigned to each 3‐month time point was the body weight closest to that time point within ±45 days. In the instance there was more than one body weight measurement for a specific 3‐month interval, the body weight that was closest to the 3‐month time point was chosen. While a 45‐day window was allowed, the selected weights were 16.7 ± 12.7 and 8.3 ± 8.2 days from the time point of interest pre‐diagnosis and diagnosis, respectively. Due to inherent differences in absolute body weight between patients and patient characteristics, percent change relative to the first body weight measured in the time frame of interest was calculated. Body mass index (BMI) was calculated and classified as underweight, normal weight, overweight or obese as per the World Health Organization criteria [14]. All charts were manually reviewed to determine intentionality of weight loss. Unintentional, cancer‐associated weight loss was based on the international consensus definition of cachexia summarized as ≥ 5% weight loss for patients with BMI ≥ 20 kg/m^2^ or ≥ 2% weight loss for patients with a BMI < 20 kg/m^2^ [3]. Only patients with measured UWL were included in the final cohort. Patients with intentional weight loss, weight loss related to fluid status, an additional primary malignancy or weight loss with unclear intention were excluded.

### 2.3 Determination and Documentation of Unintentional Weight Loss

Patients were classified into three groups based on the documentation of UWL by physicians or advanced practice providers in the electronic medical record: (1) no documentation of UWL, (2) documentation of UWL without use of an ICD diagnosis code or (3) documentation with the use of an ICD diagnosis code. Use and timing of weight loss ICD diagnosis codes were extracted from the EHR. Due to the date range for data extraction included in this cohort, both ICD‐9 and ICD‐10 codes were extracted. Code descriptions included abnormal weight loss, underweight, anorexia, cachexia and unspecified, mild, moderate and severe protein‐calorie malnutrition (Table S1). Charts were manually reviewed by two independent reviewers for documentation of UWL in free text progress notes of physicians and advanced practice providers. When assessing the relationship between stage at cancer diagnosis and percent weight loss, patients with 2.5% weight loss were included in the analyses along with patients who met the criteria for cachexia.

### 2.4 Statistical Analysis

Descriptive statistics were used to summarize patient and tumour characteristics. Student's *t*‐test or one‐way ANOVA was used to compare baseline characteristics between cachexia status groups for continuous variables. Chi‐square or Fisher's exact tests were used to test associations for categorical variables and Mann–Whitney U test or Kruskal–Wallis test for ordinal variables. Multiple logistic regression was used to predict binary UWL documentation with all patient characteristics listed in Table 1, age group (< 65 or ≥ 65 years old), primary malignancy (GI or NSCLC), stage of disease (Stages 1 and 2 or Stages 3 and 4), initial BMI (BMI < 30 kg/m^2^ or BMI ≥ 30 kg/m^2^), race (White, Black and other) and sex (male and female) as main effects. Repeated measures mixed‐effect model using the restricted maximum likelihood method was used to compare weight change over time between groups. A significance level was adjusted for multiple comparisons using Tukey adjustment. All tests were two‐sided and performed at the 5% significance level using Prism GraphPad (Version 10.1.2; GraphPad Software, La Jolla, CA) and SAS 9.4 (SAS Institute, Cary, NC).

**TABLE 1:** Patient characteristics.

	Total cohort						Gastrointestinal cancer						Non‐small cell lung cancer					
	Unintentional weight loss						Unintentional weight loss						Unintentional weight loss					
	All (*n* = 374)	None (*n* = 161)	Documented without ICD diagnosis (*n* = 98)	Documented with ICD diagnosis (*n* = 115)	*p* 3 groups^a^	*p* 2 groups	All GI (*n* = 232)	None (*n* = 94)	Documented without ICD diagnosis (*n* = 68)	Documented with ICD Diagnosis (*n* = 70)	*p* 3 groups	*p* 2 groups	All NSCLC (*n* = 142)	None (*n* = 67)	Documented without ICD diagnosis (*n* = 30)	Documented with ICD diagnosis (*n* = 45)	*p* 3 groups	*p* 2 groups
Age (years)^b^	68.9 ± 12.0	68.1 ± 11.9	68.3 ± 12.6	70.5 ± 11.6	0.21	0.26	67.6 ± 12.1	66.1 ± 12.1	67.9 ± 12.3	69.5 ± 11.8	0.20	0.11	70.9 ± 11.6	70.9 ± 11.1	69.3 ± 13.4	72.1 ± 11.3	0.59	0.97
Age group^c^					0.09	0.06					0.22	0.12					0.34	0.24
< 65 years	117 (31%)	59 (37%)	30 (31%)	28 (24%)			82 (35%)	39 (41%)	23 (34%)	20 (29%)			35 (25%)	20 (30%)	7 (23%)	8 (18%)		
> 65 years	257 (69%)	102 (63%)	68 (69%)	87 (76%)			150 (65%)	55 (59%)	45 (66%)	50 (71%)			107 (75%)	47 (70%)	23 (77%)	37 (82%)		
Gender					0.80	0.53					0.73	0.59					0.69	> 0.9999
Male	184 (49%)	76 (47%)	50 (51%)	58 (50%)			124 (53%)	48 (51%)	39 (57%)	37 (53%)			60 (42%)	28 (42%)	11 (37%)	21 (47%)		
Female	190 (51%)	85 (53%)	48 (49%)	57 (50%)			108 (47%)	46 (49%)	29 (43%)	33 (47%)			82 (58%)	39 (58%)	19 (63%)	24 (53%)		
Race					0.08	0.04					0.91	0.72					0.01	0.005
White	292 (78%)	133 (83%)	75 (77%)	84 (73%)			186 (80%)	77 (82%)	54 (80%)	55 (79%)			106 (71%)	56 (84%)	21 (70%)	29 (64%)		
Black	70 (19%)	22 (14%)	20 (20%)	28 (24%)			37 (16%)	14 (15%)	11 (16%)	12 (17%)			33 (22%)	8 (12%)	9 (30%)	16 (36%)		
Other	12 (3%)	6 (3%)	3 (3%)	3 (3%)			9 (4%)	3 (3%)	3 (4%)	3 (4%)			3 (7%)	3 (4%)	0 (0%)	0 (0%)		
Stage					0.27	0.40					0.51	0.34					0.34	0.89
1	103 (27%)	48 (29%)	27 (27%)	28 (24%)			57 (25%)	23 (24%)	17 (25%)	17 (24%)			46 (32%)	25 (37%)	10 (33%)	11 (24%)		
2	62 (17%)	25 (15%)	14 (14%)	23 (20%)			51 (22%)	22 (23%)	11 (17%)	18 (26%)			11 (8%)	3 (4%)	3 (10%)	5 (11%)		
3	64 (17%)	29 (18%)	19 (19%)	16 (14%)			36 (16%)	16 (17%)	12 (18%)	8 (11%)			28 (20%)	13 (19%)	7 (23%)	8 (18%)		
4	138 (37%)	55 (34%)	35 (36%)	48 (42%)			85 (37%)	31 (33%)	27 (40%)	27 (39%)			53 (37%)	24 (36%)	8 (27%)	21 (47%)		
Unknown	7 (2%)	7 (4%)	0 (0%)	0 (0%)			3 (1%)	3 (3%)	0 (0%)	0 (0%)			4 (3%)	4 (6%)	2 (7%)	0 (0%)		
Initial BMI (kg/m^2^)	27.0 ± 5.9	27.8 ± 6.2^b^	27.3 ± 6.7^b^	25.4 ± 4.4^a^	0.003	0.01	27.7 ± 4.6	29.1 ± 6.6^b^	27.5 ± 6.8^a^	25.9 ± 4.6^a^	0.004	0.003	25.8 ± 5.2	26.1 ± 5.1	26.9 ± 6.4	24.7 ± 4.1	0.17	0.56
BMI at Cancer Diagnosis	24.6 ± 5.4	25.6 ± 5.7^b^	24.8 ± 6.1^b^	22.9 ± 3.8^a^	0.0002	0.001	25.2 ± 5.7	26.7 ± 6.0^b^	25.0 ± 6.2^ab^	23.4 ± 4.0^a^	0.0009	0.0008	23.6 ± 4.8	24.1 ± 4.9	24.5 ± 5.9	22.3 ± 3.5	0.07	0.23

Abbreviations: body mass index (BMI), International Classification of Diseases (ICD) and Unintentional weight loss (UWL).

^a^ *p*‐values for three documentation groups compare three independent UWL documentation groups: None, Documented without ICD Diagnosis and Documented with ICD Diagnosis. *p*‐values for two documentation groups compares two independent groups: None and Documented (without and with ICD diagnosis combined).

^b^ Continuous variables are mean ± SD *p*‐value is from a one‐way ANOVA for 3 documentation groups and a Student's *t*‐test between UWL 2 documentation groups.

^c^ Categorical variables are frequency (% within column for total cohort and within row for UWL documentation group). *p*‐value is from Chi square or Fisher's exact test for categorical variables and Kruskal–Wallis test (3 documentation groups) and Mann–Whitney U (2 documentation groups) for ordinal variables between UWL.

## 3 Results

Through a UT Southwestern Medical Center Institutional Review Board approved protocol, we identified 499 patients with measured weight loss per the patient's weight history in their medical record. The focus of this paper was to retrospectively investigate the recognition and diagnosis of UWL prior to a cancer diagnosis in patients with measured UWL. Thus, patients with intentional weight loss (*n* = 123; 25%) were excluded (Table S2 and Figure S1). Of the patients who were excluded, patients with obesity were 2.6 times more likely to have intentional weight loss than patients without obesity (*p* = 0.01). After exclusion for weight loss that was intentional, 376 patients with measured UWL in the 12 months prior to cancer diagnosis were included in the final cohort (Table 1). The cohort was primarily over the age of 65 years, White race and had an equal distribution of males and females. More patients in the cohort were diagnosed with Stage 1 (27%) or Stage 4 (37%) disease than Stage 2 (17%) or Stage 3 (17%) disease. Initial BMI class was generally reflective of the United States population, with 33% of the cohort with a BMI classified as overweight and 26% with obesity. Initial BMI (26.9 ± 6.4 kg/m^2^) was significantly higher than BMI at cancer diagnosis (25.2 ± 5.7 kg/m^2^; *p* < 0.0001), as expected from a cohort of patients with UWL. This was true for GI patients (27.6 ± 6.3 kg/m^2^ initial BMI vs. 25.2 ± 5.7 kg/m^2^ at cancer diagnosis; *p* < 0.0001) and NSCLC patients (25.8 ± 5.2 kg/m^2^ initial BMI vs. 23.6 ± 4.8 kg/m^2^ at cancer diagnosis; *p* < 0.0001). The odds of having obesity (BMI > 30 kg/m^2^) were higher in GI patients than NSCLC patients (OR = 2.7 95% CI: 1.6 to 4.5; *p* = 0.0002).

**FIGURE 1:**
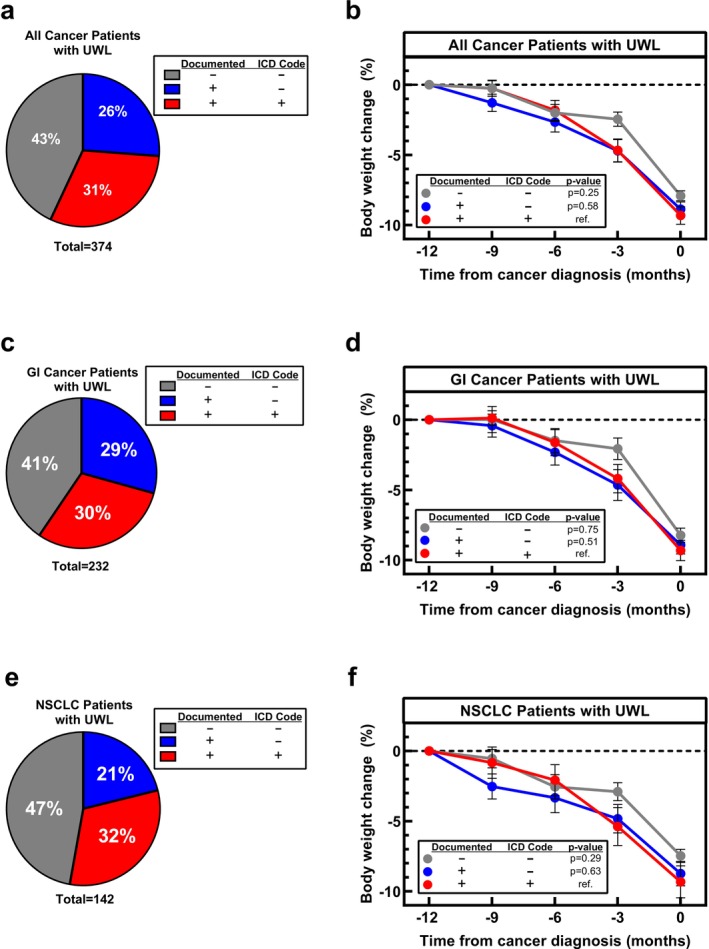
Frequency of unintentional weight loss (UWL) documentation in patients with measured UWL prior to cancer diagnosis (a). UWL documentation was not associated with primary malignancy (Fisher's Exact Test, *p* = 0.20) when assessing gastrointestinal (GI) cancer patients with UWL (c) and non‐small cell lung cancer patients (NSCLC) with UWL (e). Percent UWL did not differ between UWL documentation. This was true for all patients with UWL (b), patients with GI cancer patients with UWL (d) and NSCLC patients with UWL (f). Data (b, d and f) are shown as Mean ± SEM *p*‐values are fixed effects of repeated measures mixed‐effect model using the restricted maximum likelihood method and adjustment for multiple comparisons using Tukey adjustment to compare weight change over time between weight loss documentation groups.

We assessed the use of 8 ICD codes related to weight loss or malnutrition in this cohort of patients with measured UWL, (Table S1). Codes were assigned prior to cancer diagnosis in only 115 (31%) of the 376 patients with UWL (Figure 1a). Multiple codes may have been used for one patient prior to cancer diagnosis, with a total of 120 codes used in the 115 patients. While codes related to malnutrition were included, the most common code was abnormal weight loss used in 81% of the patients who had a UWL diagnosis prior to cancer diagnosis. Acknowledging that healthcare providers may recognize UWL but not apply an ICD diagnosis, documentation of UWL in free text progress notes was also considered. Weight loss documentation without an ICD diagnosis occurred in 98 patients (26%), resulting in a total documentation of UWL prior to cancer diagnosis occurring in 213 (57%) of patients. Total documentation of UWL was not statistically different between GI cancer (59%; Figure 1c) and NSCLC patients (53%; *p* = 0.24; Figure 1e).

To discern if documentation of UWL and use of ICD diagnosis codes were associated with a greater magnitude of weight loss, we evaluated the kinetics of weight change over 12 months preceding cancer diagnosis in patients without and with ICD documented UWL. Weight trajectories and average percent weight loss did not differ significantly between patients without documentation of UWL (7.9% ± 0.4%), documented UWL without an ICD diagnosis (8.9% ± 0.5%) and documented UWL with an ICD diagnosis (9.3% ± 0.6%; *p* = 0.21; Figure 1b). This trend was consistent in the subsets of GI cancer (*p* = 0.62; Figure 1d) and NSCLC patients (*p* = 0.24; Figure 1f). Gastrointestinal cancer patients without documentation of UWL lost an average of 8.2 ± 0.5% of their body weight pre‐cancer diagnosis, while GI cancer patients with documented UWL without an ICD diagnosis lost 8.9 ± 0.6% and GI cancer patients with an ICD diagnosis lost 9.3 ± 0.7% of their body weight pre‐cancer diagnosis. NSCLC cachexia patients without documented UWL lost an average of 7.5 ± 0.5% of their initial body weight pre‐cancer diagnosis compared to NSCLC cachexia patients with documented UWL without an ICD diagnosis who lost 8.7 ± 0.9% and NSCLC patients with an ICD diagnosis who lost 9.3 ± 1.1% of their body weight pre‐cancer diagnosis. In the 213 patients with documented UWL diagnosis, the documentation of UWL occurred approximately 7 months after their initial weight loss. Even in patients with documented UWL, it was not recognized until the patients had lost an average of 7.5% of their initial body weight.

We then investigated if UWL documentation differed between subpopulations of cancer cachexia patients. Univariate analysis revealed UWL documentation was not associated with sex (*p* = 0.80; Table 1). When patients were dichotomized as < 65 years and ≥ 65 years of age, UWL was documented in 155 (60%) of patients ≥ 65 years old (Figure S2c) compared to 58 (50%) in patients < 65 years old (Figure S2a). However, this association did not reach statistical significance (*p* = 0.06). Weight trajectories and percent UWL did not differ between patients without and with documentation of UWL within patients < 65 years (*p* = 0.58; Figure S2b) and ≥ 65 years of age (*p* = 0.33; Figure S2d). In all cancer patients with UWL, race was associated with UWL documentation (*p* = 0.04; Table 1). However, subanalysis revealed, race was significantly associated with UWL documentation in NSCLC cachexia patients (*p* = 0.005) with UWL documented in 76% of Blacks compared to 47% of Whites and 0% of other minorities. In all cancer patients with UWL, documentation of UWL was not associated with stage of disease at cancer diagnosis (*p* = 0.40).

**FIGURE 2:**
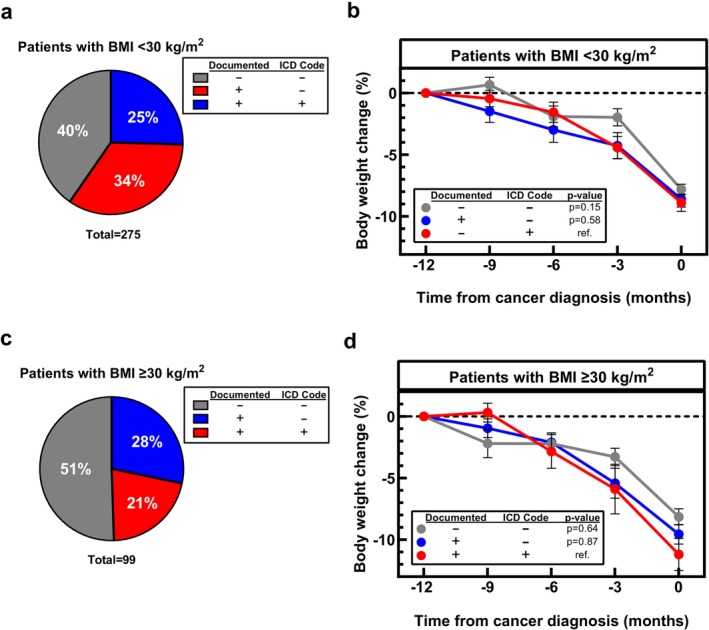
Obesity status and frequency of unintentional weight loss (UWL) related to UWL diagnosis in patients with measured weight loss pre‐cancer diagnosis. Frequency of UWL diagnosis in patients without obesity (body mass index, BMI < 30 kg/m^2^; a) and patients with obesity (BMI ≥ 30 kg/m^2^; c). UWL documentation was trending toward associated with obesity status (Fisher's Exact Test, *p* = 0.05). Percent UWL did not differ between patients without and with a UWL diagnosis. This was true for patients without (b) and with obesity (d). Data (b and d) are shown as Mean ± SEM *p*‐values are fixed effects of repeated measures mixed‐effect model using the restricted maximum likelihood method and adjustment for multiple comparisons using Tukey adjustment to compare weight change over time between UWL documentation groups.

Both initial and cancer diagnosis BMI were consistently lower in patients with documented UWL compared to cancer cachexia patients without documented UWL (all *p* < 0.005). Therefore, we compared documentation of UWL in patients with obesity (initial BMI ≥ 30 kg/m^2^) to patients without obesity (initial BMI < 30 kg/m^2^). While documentation of UWL was not associated with obesity (*p* = 0.10), ICD diagnosis codes were used more frequently in cancer patients without obesity (34%; Figure 2a) than with obesity (21%; *p* = 0.02; Figure 2c). Percent weight loss in the 12 months prior to cancer diagnosis did not differ between patients without obesity and with obesity (*p* = 0.48). In cancer patients without obesity, patients without documented UWL lost 7.8 ± 0.4% of their body weight pre‐cancer diagnosis compared to patients with documented UWL without an ICD diagnosis code who lost 8.6 ± 0.6% and with an ICD diagnosis code who lost 8.9 ± 0.7% of their body weight (*p* = 0.84; Figure 2b). In cancer patients with obesity, patients without documented UWL lost 8.1 ± 0.7% of their initial body weight compared to patients with documented UWL without an ICD diagnosis who lost 9.6 ± 0.8% and with an ICD diagnosis who lost 11.2 ± 1.3% of their body weight (*p* = 0.89; Figure 2d). When patient characteristics were included in logistic regression analysis, age 65 years and older (OR = 1.82 95% CI: 1.15 to 2.90; *p* = 0.01), BMI < 30 kg/m^2^ (OR = 1.78 (95% CI:1.09 to 2.92); *p* = 0.02), having a GI cancer (OR = 1.65 95% CI: 1.05 to 2.61; *p* = 0.03) and Black race (OR = 2.16 95% CI: 1.22 to 3.92; *p* = 0.008) were statistically significant determinants of documentation of UWL pre‐cancer diagnosis (Table 2).

**TABLE 2:** Multiple logistic regression analysis of unintentional weight loss documentation^a^.

Variable	Odds ratio	*p*
Age		
< 65 years old	Ref	
≥ 65 years old	1.82 (95% CI: 1.15 to 2.90)	**0.0112**
Primary malignancy		
NSCLC	Ref	
GI cancer	1.65 (95% CI: 1.05 to 2.61)	**0.0308**
Stage at diagnosis		
Early (Stages 1 and 2)	Ref	
Late (Stages 3 and 4)	1.13 (95% CI: 0.78 to 1.74)	0.5682
Initial BMI		
BMI ≥ 30 kg/m^2^	Ref	
BMI < 30 kg/m^2^	1.78 (95% CI: 1.09 to 2.92)	**0.0219**
Race		
White	Ref	
Black	2.16 (95% CI: 1.22 to 3.92)	**0.0079**
Other	0.92 (95% CI: 0.27 to 3.18)	0.8924
Sex		
Male	Ref	
Female	0.77 (95% CI: 0.50 to 1.19)	0.2390

*Note:* Categorical independent variables included in the model were as shown in the table (age, primary malignancy, stage at diagnosis, initial BMI, race and sex).

^**a**^ Documentation of unintentional weight loss either in a doctor or advanced practice providers progress note or use of an UWL International Classification of Disease (ICD) code was considered a positive outcome (dependent variable).

**FIGURE 3:**
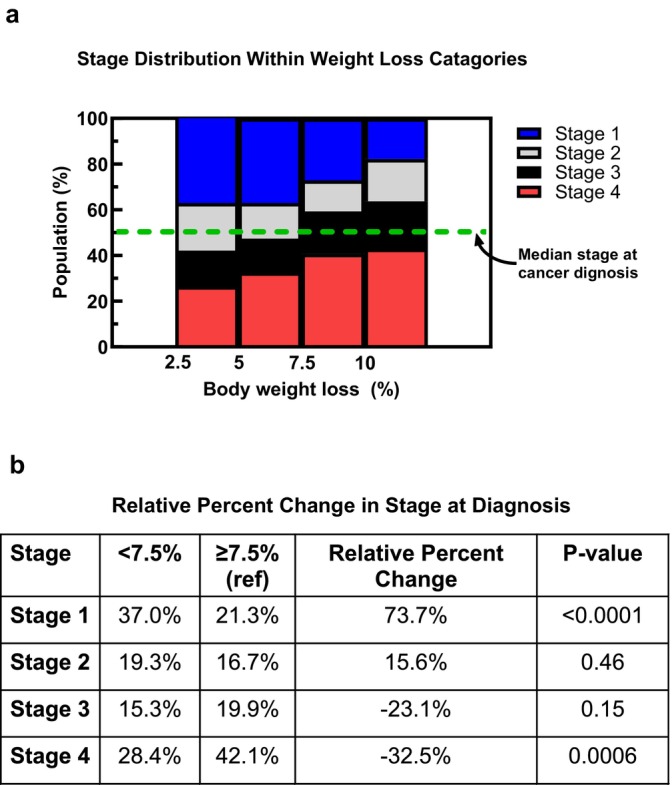
Cancer stage distribution within weight loss categories (a). Cancer stage is associated with magnitude of weight loss (*p* = 0.03; Kruskal–Wallis test). Median stage at diagnosis differed between patients who lost ≥ 7.5% (Stage 3) and < 7.5% body weight (Stage 2; *p* < 0.0001; Mann–Whitney test). Earlier identification of UWL and reducing total body weight lost would be expected to lead to an increase in patients with more treatable Stage 1 disease and a reduction in patients with terminal Stage 4 disease (b).

To determine how UWL relates to stage at cancer diagnosis, we grouped body weight loss into 4 categories, 2.5 to 4.9%, 5 to 7.4%, 7.5 to 9.9% and ≥ 10% body weight loss. Magnitude of UWL was associated with cancer stage at diagnosis (*p* < 0.0001). Patients who experienced greater than 7.5% UWL pre‐diagnosis had a median cancer stage of 3, whereas patients who experienced less than 7.5% UWL had a median cancer Stage of 2 at diagnosis (Figure 3a,b). Relative to patients with ≥ 7.5% UWL, the < 7.5% weight loss group had 73.7% more patients with Stage 1 disease (*p* < 0.0001) and 32.5% fewer patients with Stage 4 disease (*p* = 0.0006).

## 4 Discussion

Irrespective of aetiology, UWL is nearly always associated with poorer patient outcomes [9, 15]. Prompt identification of UWL can lead to earlier diagnoses and interventions for cancer and other serious illnesses. Therefore, in a retrospective cohort of cancer patients with measured weight loss we quantified the documentation of UWL by clinicians. Documentation or diagnosis of UWL was missed in nearly half of the patients with clinically significant weight loss prior to cancer diagnosis.

Missed documentation of UWL was consistent among all cancer patients with UWL. When adjusted for other patient characteristics, there was a statistically significant association between UWL documentation and age suggesting that clinicians are more sensitive to UWL in older patients. UWL is more common in older adults and is associated with increased morbidity and mortality, particularly in patients with a lower BMI and increased frailty [5]. The American Society of Clinical Oncology recognizes the importance of geriatric assessment in patients older than 65 years. While clinicians report frequent assessment of functional status and falls, tools for assessing weight loss are underutilized [16]. Normal age‐related weight loss is reported to only be 0.1 to 0.2 kg per year [17, 18]. Therefore, rapid UWL aetiology in the elderly should be investigated and not deemed a natural process of aging.

Multiple logistic regression analysis also revealed documentation of UWL was missed more often in patients with obesity (BMI ≥ 30 kg/m^2^) compared to patients without obesity (BMI < 30 kg/m^2^). Indeed, the odds of UWL documentation were over 150% greater in patients without obesity compared to patients with obesity despite similar percent UWL and greater absolute weight loss in patients with obesity. This is particularly concerning as increased adiposity is associated with an increased risk for multiple primary malignancies including gastrointestinal cancers resulting in more patients having obesity and cancer [19, 20, 21]. Missed documentation of UWL in patients with a BMI ≥ 30 kg/m^2^ may be related to the perception and desire for weight loss in patients with obesity. While not investigated herein, implicit and explicit weight bias and weight stigma can influence a healthcare provider's clinical decision making and diagnostic and treatment recommendations [22]. These biases may alter the provider's and the patient's perception and interpretation of UWL in patients with obesity. The results also demonstrated an association between recognition of UWL and race. The odds of UWL recognition were higher in Black patients than in Whites. This may be due in part to the perceptions of weight status and weight loss differences between racial and ethnic groups [23].

Furthermore, the magnitude of UWL did not influence clinicians to document UWL. In the 50% of patients with recognized weight loss, UWL was documented 7 months after the initiation of the UWL. By the time UWL was documented, patients had already lost ~7.5% of their body weight, far exceeding the diagnostic threshold for cachexia. Therefore, UWL documentation did not lead to earlier cancer staging at the time of cancer diagnosis. However, cancer stage at diagnosis was lower in patients with UWL < 7.5% with a 70% increase with Stage 1 disease. Therefore, earlier identification of UWL and reducing total body weight lost would be expected to lead to an increase in patients with more treatable Stage 1 disease and a reduction in patients with terminal Stage 4 disease.

We acknowledge study limitations analogous to all retrospective analyses of EHR data. The data extracted were intended to guide clinical care and were not initially collected with the rigour ideal for research. We were limited to associative relationships, with the ultimate aetiology behind weight change being correlative. In addition, the findings within this cohort may not extrapolate to other cohorts. Specifically, our data suggest patient race may influence recognition of UWL. However, the racial diversity of this cohort is limited and investigation in more racially diverse cohorts is needed to better understand the nuance of UWL documentation in other cultures.

Despite these limitations, monitoring and addressing weight loss in the primary and specialized care settings is of the utmost importance for early cancer diagnosis and improved patient outcomes. However, body weight is one of many clinical variables that require review by healthcare providers and is easily overlooked by both patients and clinicians. Under‐documented UWL is not specific to this cohort of patients. In a cohort of 290 patients with UWL of mixed aetiology, only 21% were diagnosed with UWL [24]. Similarly, in a retrospective chart review of 100 000 adult patients seen in outpatient clinics over a five‐year period, 170 randomly‐selected weight loss periods were reviewed. Only 39% of the weight loss periods were recognized by the clinician at the index visit and an additional 1% at the next primary care visit [25]. While the misidentification of UWL is likely due to multiple factors, it is difficult for patients and clinicians to identify UWL, particularly at the early stages where early intervention could improve clinical outcomes.

These data support the need for technology‐assisted identification of weight loss and consideration of weight loss in clinical decision making. With the interoperability of EHR, the responsibility of weight monitoring does not fall on one healthcare practitioner. Routine weight monitoring is more manageable than ever as body weight data from various clinical encounters can be aggregated to decipher weight change over time. Furthermore, development and utilization of automated and artificial intelligence‐assisted analyses of CT scans and ultrasounds and the development of wearable bioelectronic devices enable longitudinal clinical evaluation of muscle atrophy, a stronger predictor of patient survival and quality of life than weight loss alone [26, 27, 28, 29, 30, 31, 32]. The implementation of an automatic flag for weight loss in the EHR that prompts further investigation through age directed cancer screenings is a powerful strategy that could mitigate the impact of human error and biases in the identification and workup of UWL. This strategy will increase the ease at which UWL is used as complementary screening criteria for cancer leading to earlier diagnosis and improved patient outcomes.

## Conflicts of Interest

P.I. has worked with AstraZeneca in an advisory capacity and received funding from Incyte unrelated to this work. R.E.I. laboratory has received funding from Pfizer Inc. and Incyte unrelated to this work. The other authors declare no conflicts of interest.

## Supporting information


**Table S1:** International Classification of Diseases (ICD) codes related to weight loss or malnutrition used prior to cancer diagnosis.
**Table S2:** Weight loss aetiology.
**Figure S1:** Consort diagram of patients included in the study. Cancers include gastrointestinal (GI) cancers (gastroesophageal, colorectal, hepatobiliary, pancreatic cancers) and non‐small cell lung cancer (NSCLC). Patients with measured unintentional weight loss (UWL) were included in the final cohort (*n* = 374). Electronic medical records were assessed for the use of UWL and malnutrition related International Classification of Diseases (ICD) codes. Patients were categorized into three groups, (1) patients without documented UWL (*n* = 161; 43%), (2) patients with documentation of UWL by physician or advanced practice provider but no UWL ICD diagnosis (*n* = 98; 26%) and (3) ICD diagnosis of UWL (*n* = 115; 31%).
**Figure S2:** Age group and frequency of unintentional weight loss (UWL) diagnosis in patients with measured UWL pre‐cancer diagnosis. Frequency of UWL documentation in patients < 65 years old (A) and patients ≥ 65 years old. UWL documentation was not associated with age group (Fisher's exact test, *p* = 0.09). Percent UWL did not differ between documentation. This was true for patients < 65 years old (C) and patients ≥ 65 years old (D). Data (B and D) are shown as Mean ± SEM *p*‐values are fixed effects of repeated measures mixed‐effect model using the restricted maximum likelihood method and adjustment for multiple comparisons using Tukey adjustment to compare weight change over time between UWL documentation groups.
