# Supplementary material for: Undetected Weight Loss Associates With Upstaging in Cancer Patients
Source: J Cachexia Sarcopenia Muscle. 2026 Apr 8;17(2):e70266. doi: 10.1002/jcsm.70266 (PMC13058569; doi:10.1002/jcsm.70266)
Supplement: Supplementary file 1 — Table S1: International Classification of Diseases (ICD) codes related to weight loss or malnutrition used prior to cancer diagnosis. Table S2: Weight loss aetiology. Figure S1: Consort diagram of patients included in the study. Cancers include gastrointestinal (GI) cancers (gastroesophageal, colorectal, hepatobiliary, pancreatic cancers) and non‐small cell lung cancer (NSCLC). Patients with measured unintentional weight loss (UWL) were included in the final cohort (n = 374). Electronic medical records were assessed for the use of UWL and malnutrition related International Classification of Diseases (ICD) codes. Patients were categorized into three groups, (1) patients without documented UWL (n = 161; 43%), (2) patients with documentation of UWL by physician or advanced practice provider but no UWL ICD diagnosis (n = 98; 26%) and (3) ICD diagnosis of UWL (n = 115; 31%). Figure S2: Age group and frequency of unintentional weight loss (UWL) diagnosis in patients with measured UWL pre‐cancer diagnosis. Frequency of UWL documentation in patients < 65 years old (A) and patients ≥ 65 years old. UWL documentation was not associated with age group (Fisher's exact test, p = 0.09). Percent UWL did not differ between documentation. This was true for patients < 65 years old (C) and patients ≥ 65 years old (D). Data (B and D) are shown as Mean ± SEM p‐values are fixed effects of repeated measures mixed‐effect model using the restricted maximum likelihood method and adjustment for multiple comparisons using Tukey adjustment to compare weight change over time between UWL documentation groups. [file JCSM-17-e70266-s001.docx]

**Supplemental Figures and Tables**

**Supplemental Table 1.** International Classification of Diseases (ICD) codes related to weight loss or malnutrition used prior to cancer diagnosis.

| **Description (ICD-10)** | **ICD-9** | **ICD-10** | **Frequency of use** |
| --- | --- | --- | --- |
| Abnormal weight loss | 783.21 | R63.4 | 93 |
| Anorexia | 783 | R63.0 | 10 |
| Unspecified severe protein-calorie malnutrition | 262 | E43 | 10 |
| Unspecified protein-calorie malnutrition | 263.8 | E46 | 4 |
| Cachexia | 799.4 | R64 | 2 |
| Moderate protein-calorie malnutrition | 263 | E44.0 | 1 |
| Mild protein-calorie malnutrition | 263.1 | E44.1 | 0 |
| Underweight | 783.22 | R63.6 | 0 |
| **Total** |  |  | **120** |

**Supplemental Table 2.** Weight loss etiology

| **Reason for weight loss** | **All Patients** | **Underweight** | **Normal Weight** | **Overweight** | **Obesity** |
| --- | --- | --- | --- | --- | --- |
| Unintentional | 374 (75%) | 17 (94%) | 135 (89%) | 125 (73%) | 99 (63%) |
| Lifestyle changes or weight loss medication | 38 (8%) | 0 | 1 (1%) | 13 (8%) | 24 (15%) |
| Fluid related (on diuretic) | 37 (7%) | 0 | 6 (4%) | 19 (11%) | 12 (8%) |
| Receiving cancer treatment for another primary malignancy | 35 (7%) | 1 (6%) | 8 (5%) | 12 (7%) | 14 (9%) |
| Unable to determine intention with confidence | 15 (3%) | 0 | 2 (1%) | 2 (1%) | 9 (6%) |
| **Total** | **499** | **18** | **152** | **171** | **158** |

**Supplemental Figure 1**

**
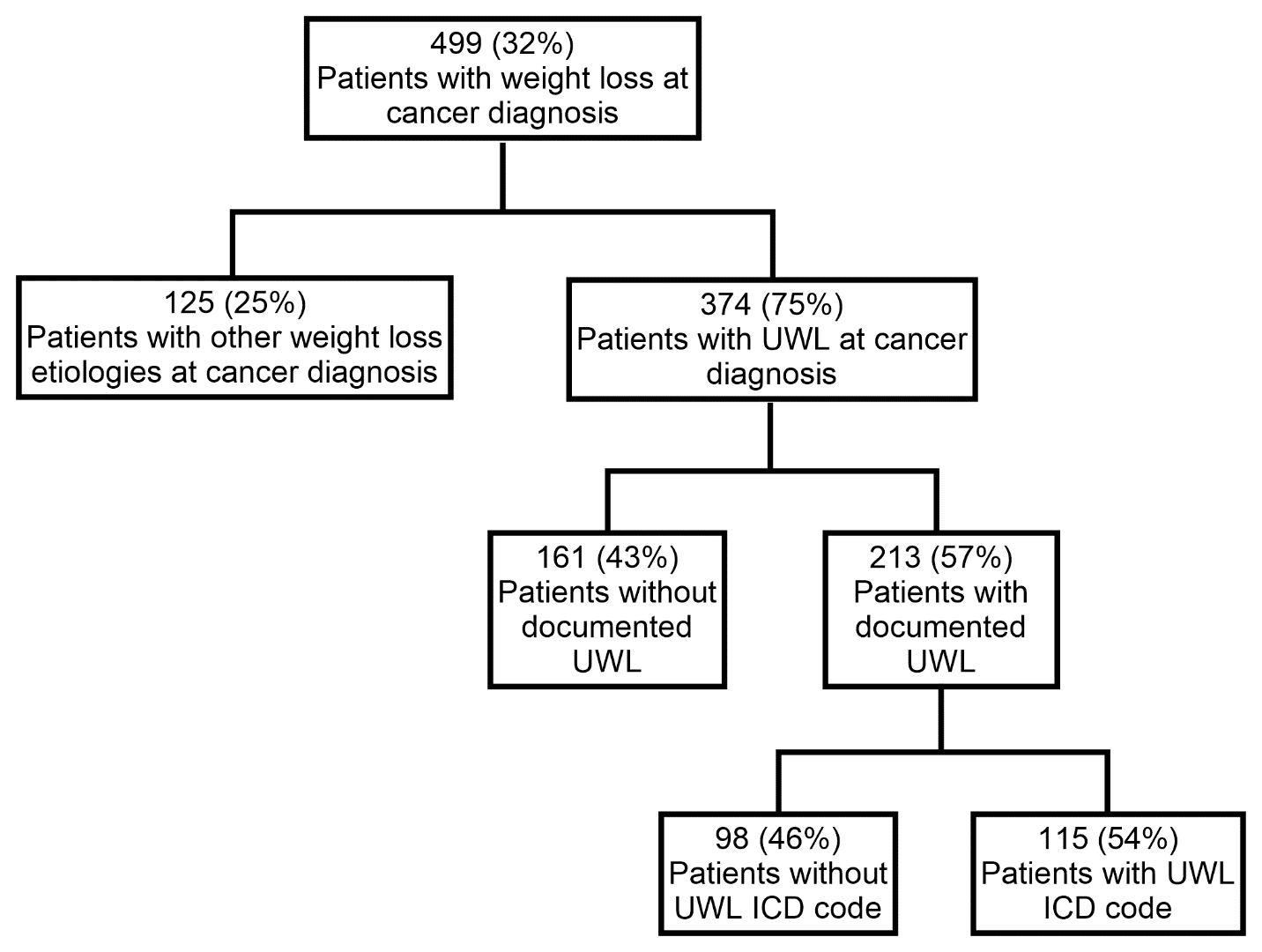
**

**Supplemental Figure 1** Consort diagram of patients included in the study. Cancers include gastrointestinal (GI) cancers (gastroesophageal, colorectal, hepatobiliary, pancreatic cancers) and non-small cell lung cancer (NSCLC). Patients with measured unintentional weight loss (UWL) were included in the final cohort (n=374). Electronic medical records were assessed for the use of UWL and malnutrition related International Classification of Diseases (ICD) codes. Patients were categorized into 3 groups, 1) patients without documented UWL (n=161; 43%), 2) patients with documentation of UWL by physician or advanced practice provider but no UWL ICD diagnosis (n=98; 26%), and 3) ICD diagnosis of UWL (n=115; 31%).

**Supplemental Figure 2.**

**
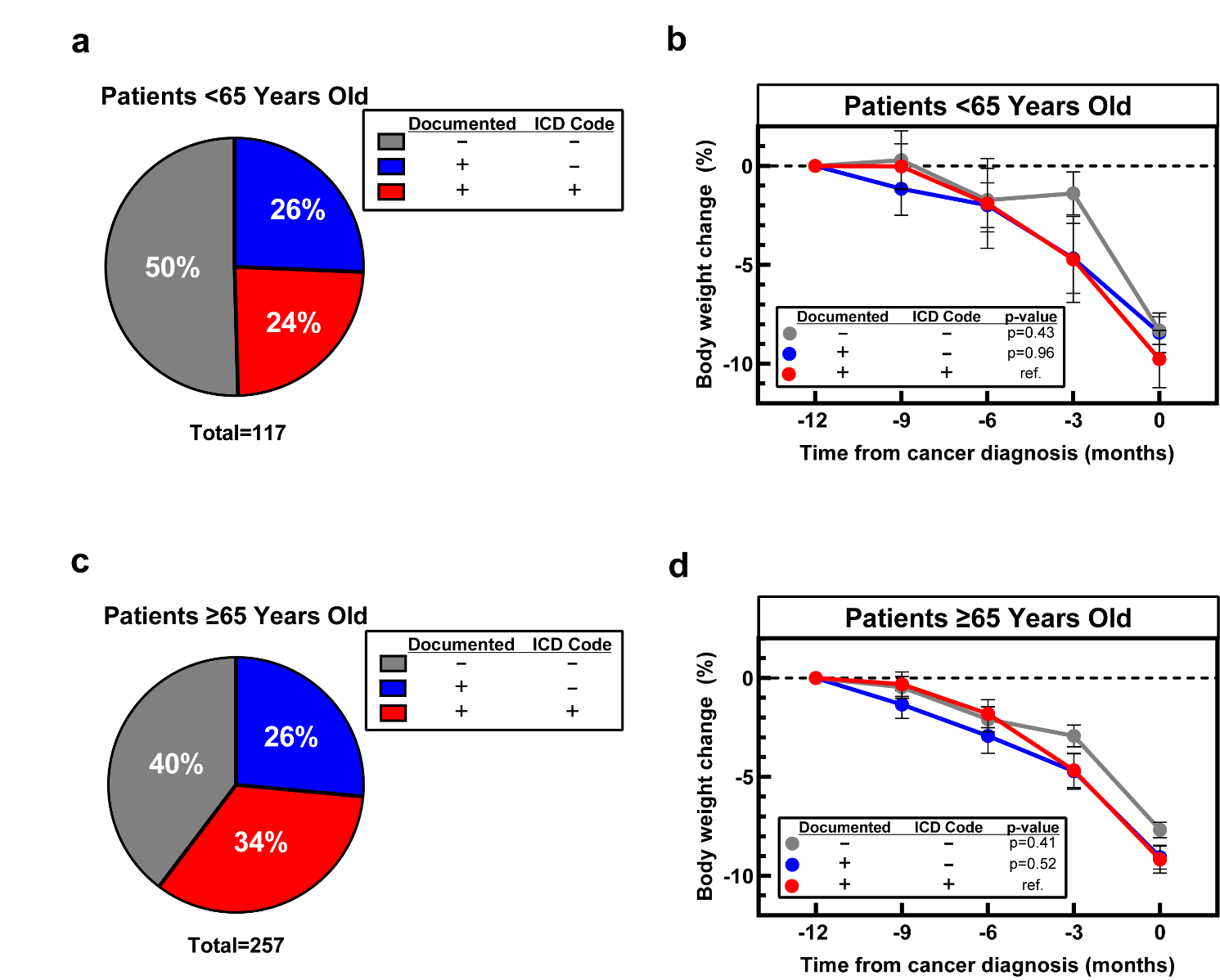
**

**Supplemental Figure 2.** Age group and frequency of unintentional weight loss (UWL) diagnosis in patients with measured UWL pre-cancer diagnosis. Frequency of UWL documentation in patients <65 years old (A) and patients ≥65 years old. UWL documentation was not associated with age group (Fisher’s exact test, p=0.09). Percent UWL did not differ between documentation. This was true for patients <65 years old (C) and patients ≥65 years old (D). Data (B and D) are shown as Mean ± SEM. P-values are fixed effects of repeated measures mixed-effect model using the restricted maximum likelihood method and adjustment for multiple comparisons using Tukey adjustment to compare weight change over time between UWL documentation groups.
